# COVID-19 and beyond: leveraging artificial intelligence for enhanced outbreak control

**DOI:** 10.3389/frai.2023.1266560

**Published:** 2023-11-08

**Authors:** Faiza Farhat, Shahab Saquib Sohail, Mohammed Talha Alam, Syed Ubaid, Shakil, Mohd Ashhad, Dag Øivind Madsen

**Affiliations:** ^1^Department of Zoology, Aligarh Muslim University, Aligarh, India; ^2^Department of Computer Science and Engineering, Jamia Hamdard, New Delhi, India; ^3^Faculty of Electronic and Information Technology, Warsaw University of Technology, Warsaw, Poland; ^4^USN School of Business, University of South-Eastern Norway, Hønefoss, Norway

**Keywords:** COVID-19, novel coronavirus, artificial intelligence, Monkeypox, ChatGPT

## Abstract

COVID-19 has brought significant changes to our political, social, and technological landscape. This paper explores the emergence and global spread of the disease and focuses on the role of Artificial Intelligence (AI) in containing its transmission. To the best of our knowledge, there has been no scientific presentation of the early pictorial representation of the disease's spread. Additionally, we outline various domains where AI has made a significant impact during the pandemic. Our methodology involves searching relevant articles on COVID-19 and AI in leading databases such as PubMed and Scopus to identify the ways AI has addressed pandemic-related challenges and its potential for further assistance. While research suggests that AI has not fully realized its potential against COVID-19, likely due to data quality and diversity limitations, we review and identify key areas where AI has been crucial in preparing the fight against any sudden outbreak of the pandemic. We also propose ways to maximize the utilization of AI's capabilities in this regard.

## 1. Introduction

**Figure 1:**
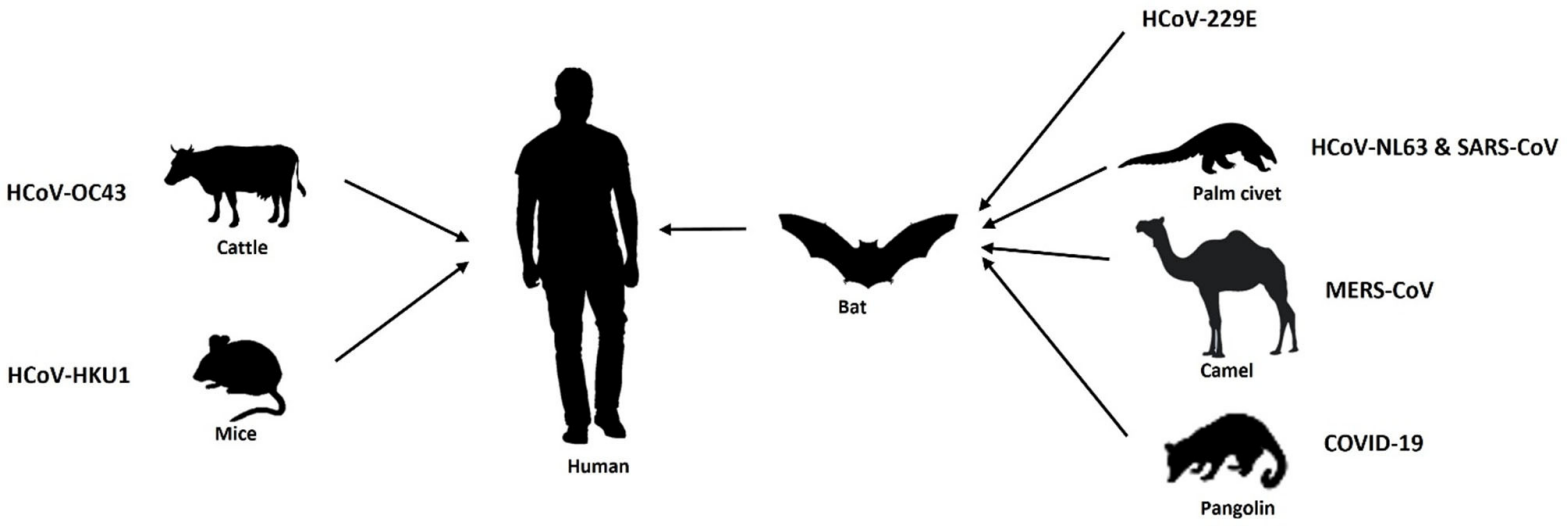
Zoonotic transmission of Coronavirus diseases.

Throughout history, our world has confronted several pandemics, including the Spanish flu of 1918, the HIV/AIDS crisis in the 1980s, and the H1N1 influenza pandemic in 2009. These events have posed significant challenges to healthcare systems and spurred innovative responses. The World Health Organization (WHO) along with different governmental administrators have come together periodically to battle against these pandemics so far. In recent times, the COVID-19 pandemic has underscored the importance of advanced technologies in disease control. Coronaviruses are RNA viruses that belong to the coronaviridae family, having four reported genera, i.e., alpha (α), beta (β), gamma (γ), and delta (δ). Among which alpha and beta coronaviruses infect mammals, whereas gamma and delta coronaviruses infect birds. Further, α-coronaviruses comprise human coronaviruses 229E (HCoV 229E) and NL63 (HCoV-NL63), while β-coronaviruses constitute human coronaviruses such as HKU1, OC43, Middle East respiratory syndrome coronavirus (MERS-CoV-2) and Severe Acute Respiratory Syndrome Coronavirus (SARS-CoV). The currently emerging novel coronavirus SARS-CoV-2 causing COVID-19 has also been classified in the genera of β-coronaviruses. Almost all types of coronaviruses show zoonosis (transmission through a vector host/reservoir) or, have an intermediate host including SARS CoV-2. Zoonotic transmissions of Coronavirus infection are depicted in Figure 1 (Chan-Yeung and Xu, 2003; Alimadadi et al., 2020).

Artificial Intelligence (AI) and Machine Learning (ML) have proved to be a boon in the battle against COVID-19. AI-driven tools have been used for early detection by analyzing large sets of medical data, tracking the virus's spread, optimizing resource allocation, and expediting vaccine development. Machine learning and data analysis, facilitated by AI, have provided valuable insights into virus behavior and epidemiological trends, aiding decision-makers in implementing effective strategies. AI's contributions have not only improved our ability to respond to crises but have also reshaped our approach to public health and disease management in the modern era of research and healthcare. Various researchers have proposed that ML algorithms can be employed to integrate and analyze the extensive data of COVID-19 victims. This provides insight into the virus' propagation pattern, which potentially increases the diagnosis rate and efficiency. It also assists in the development of new clinically effective methods which identify the most vulnerable patients based on their unique genetic and physiological characteristics (Alimadadi et al., 2020). This study reviews the existing state of AI applications while battling COVID-19, driven by the need to underline the importance of deploying AI in resisting the COVID-19 disaster. We present a comprehensive analysis of studies on the virus' overall impact, as well as the technology used to combat the COVID-19 pandemic (Chan-Yeung and Xu, 2003).

## 2. Background

We have witnessed a couple of major developments in the preceding decades where the transmission of beta corona viruses from animals to human beings has resulted in critical illness. The initial occurrence of such a disease was 18 years back in 2002, when a novel β coronavirus originated in bats transmitted over to humans through palm civets prevalent in China, especially in the Guangdong region. This was characterized as severe acute respiratory syndrome coronavirus (SARS-CoV) and resulted in 916 casualties in China and Hong Kong (Chan-Yeung and Xu, 2003). Later in the year 2012 another β coronavirus, i.e., Middle East Respiratory Syndrome (MERS) coronavirus emerged in Saudi Arabia acquired through dromedary camels to humans and caused 858 fatalities (Memish et al., 2020). In December 2019, a number of unknown cases of pneumonia like symptoms were identified in Wuhan, China. After investigations, it was reported to be a strain of novel coronavirus SARS-CoV (SARS-CoV-2) (Wang L. S. et al., 2020) causing the coronavirus disease later termed as “COVID-19” by the World Health Organization (WHO). Though having a low mortality rate, the current strain, SARS-CoV-2, was found to be highly infectious and transmissible as compared to the other two coronaviruses viz. SARS-CoV and MERS, thus posing a major challenge for inhibiting and treating this viral infection.

**Figure 2:**
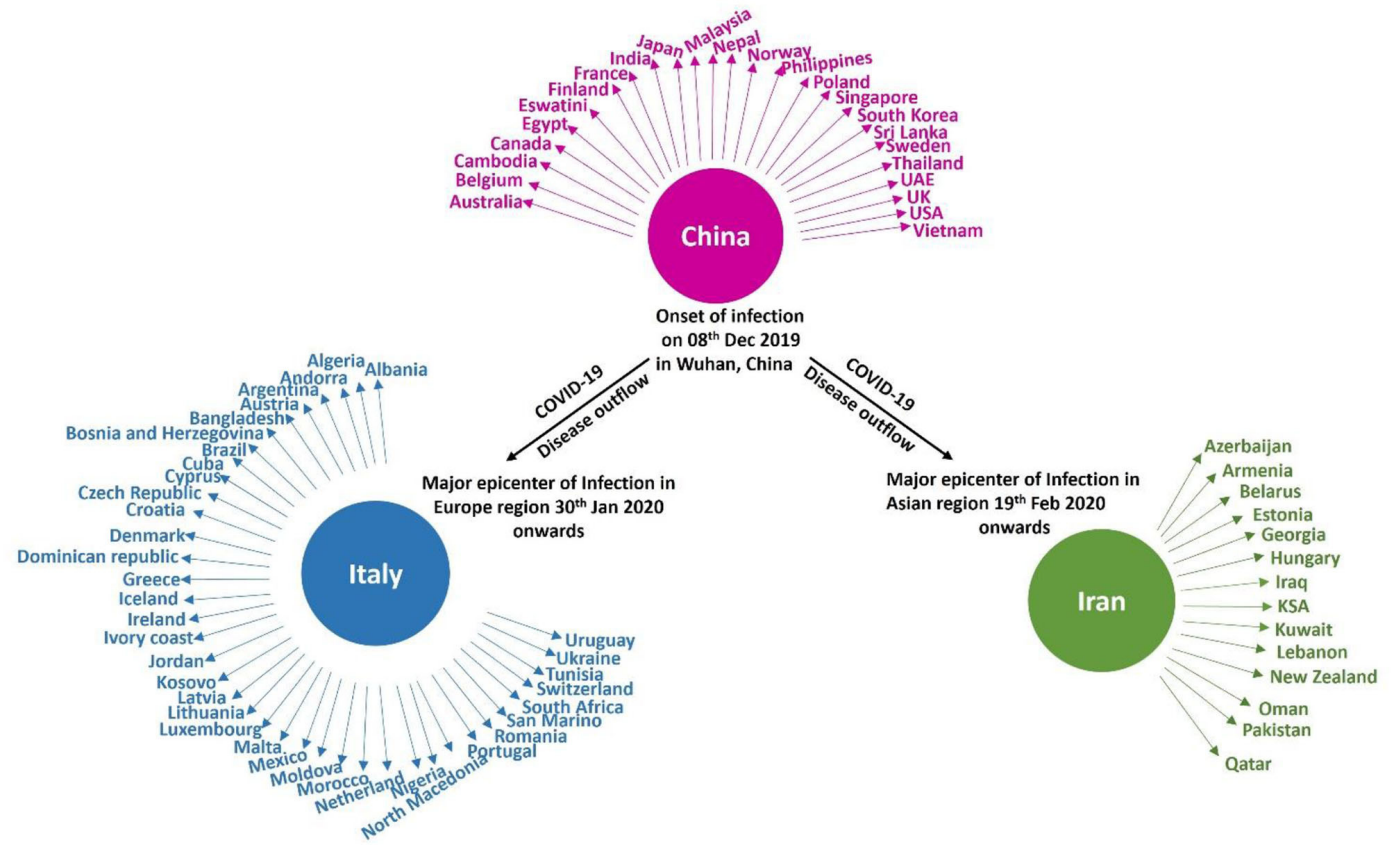
Epidemiological flow of COVID-19 from China to different parts of the world.

On February 29th, 2020, the number of cases reported of SARS-CoV-2 infection had rapidly reached up to 79,394, while the number of deaths recorded peaked up to 2,838 within a very short span of 2–3 months since the first case was recorded (Sun et al., 2020). Afterwards, it rapidly transmitted through China, Italy, and Iran, epicenters of Asia, Europe, and Middle East and, respectively, to all over the world. Figure 2 indicates the epidemiological flow of COVID-19 from China to other places in the world (Sun et al., 2020). As of June 2020, the total number of reported cases worldwide were 173 and 3.7 million people have succumbed to death till then. This article reviews and presents a brief insight into the advancements in studies, particularly concerning artificial intelligence, that have taken place over time.

### 2.1. Review method

The primary goal of this study was to determine the full scope of AI's impact on COVID-19 containment. Scopus was chosen as the first source because it contains papers from prestigious journals and conference proceedings, providing a diverse sample of the importance of AI in COVID-19 containment. Scopus search returned 332 articles for first search “artificial intelligence for medical imaging to combat coronavirus disease” keywords used are, (“ai” OR “artificial AND intelligence” OR “artificial AND intelligent” OR “machine AND learning” OR “deep AND learning”) AND (“medical” AND ima^*^) AND (“COVID-19” OR “novel AND coronavirus” OR “COVID-19 AND pandemic” OR “coronavirus” OR “SARS-CoV-2”).

Scopus search for second search “AI in managing social distancing” in the article title, abstract, or keywords, (“ai” OR “artificial AND intelligence” OR “artificial AND intelligent” OR “machine AND learning” OR “deep AND learning”) AND (social AND dist^*^) AND (“COVID-19” OR “novel AND coronavirus” OR “COVID-19 AND pandemic” OR “coronavirus” OR “SARS-CoV-2”), returned 159 articles. Scopus returned in total 491 articles; those were then manually reviewed for relevance to our review concept. All publications that met the following requirements were deemed relevant.

#### 2.1.1. Inclusion/exclusion criteria

**Figure 3:**
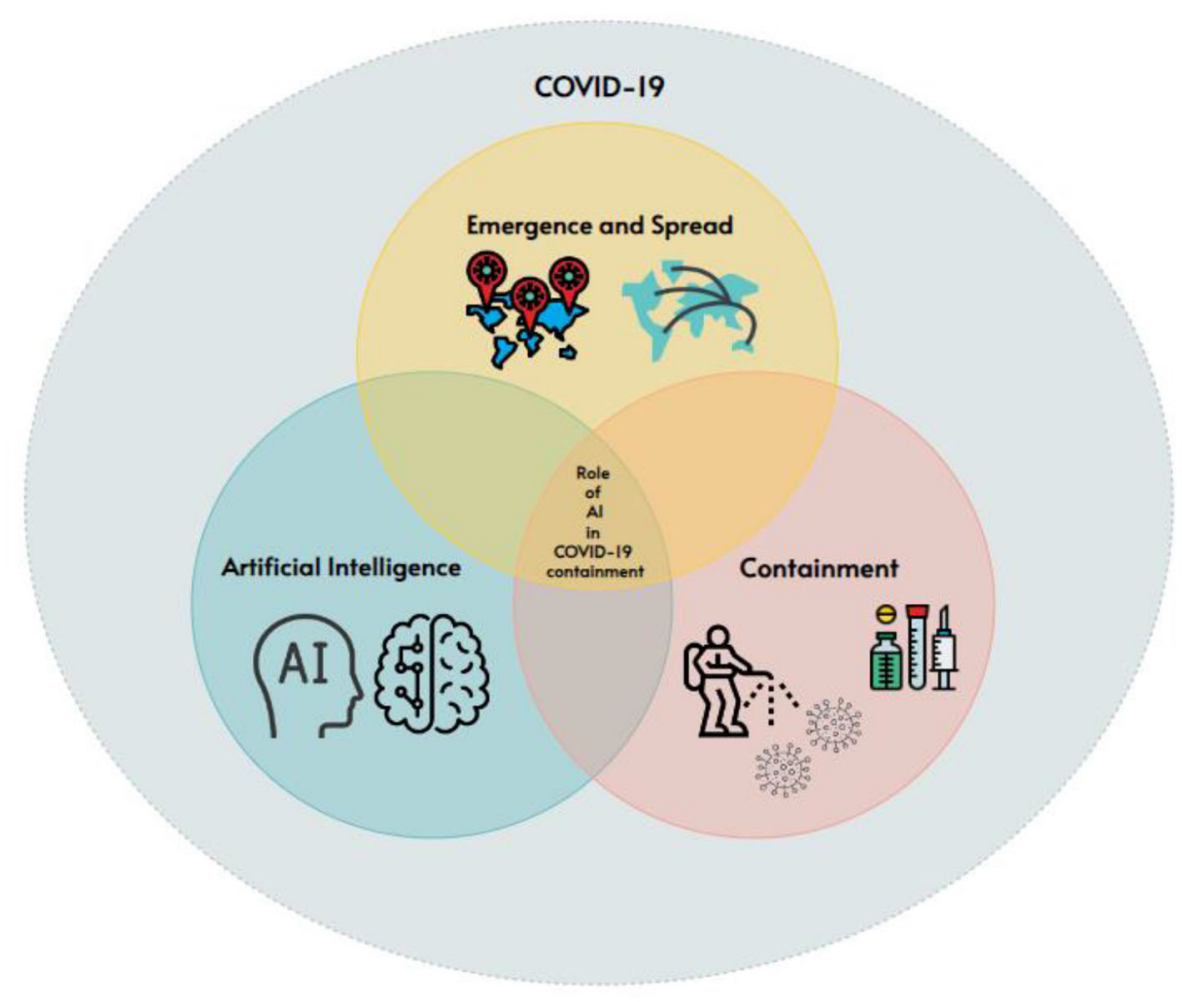
Selection criteria of papers for review.

**Figure 4:**
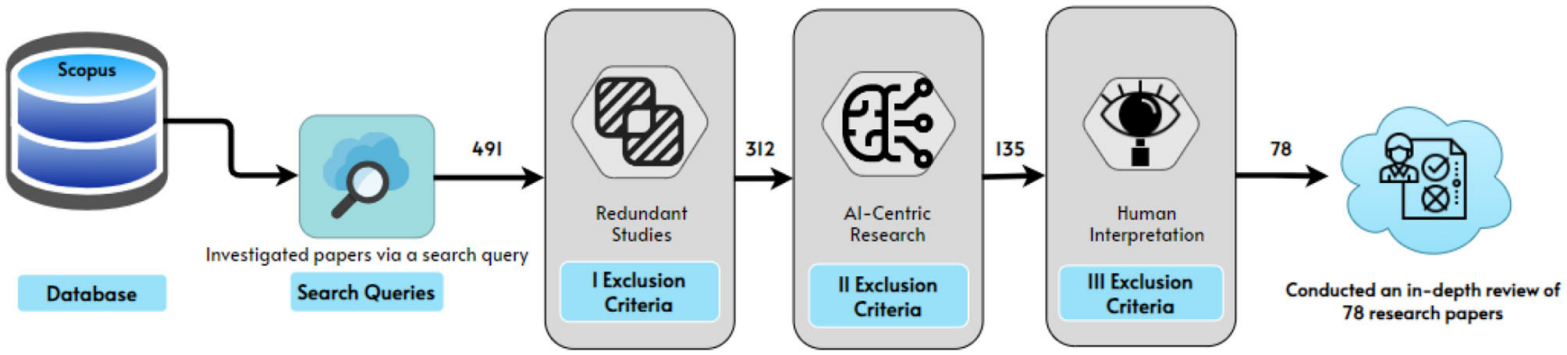
Pipeline for the selection criteria of papers.

- Literature till August 2023 that was available in English is included and unobtainable or duplicate works of literature are excluded.
- Study cases involve several topics, such as the emergence and spread of COVID-19. The present study primarily focuses on AI and its applications during the pandemic, such as data gathering systems for COVID-19, screening patients, and diagnosing, early detection of COVID-19, contact tracing, speeding up drug development, the advent of tele dermatology, and prediction of future pandemics.
- The titles and abstracts of the publications were subjected to the relevancy criterion. When the title and abstract alone were insufficient to determine if the publication met the criteria, the entire article was scanned and then the decision on inclusion or exclusion was made. This scrutiny requires specialist investigation as per the strategy as mentioned in the Figure 3. Two of the co-authors dedicatedly performed this job.
- As a result of this approach, 78 papers were selected for inclusion in the next research steps as shown in Figure 4. The first author applied the relevant criteria, while the second author randomly selected 10% of the publications for internal validity review. The second author worked independently from the first author, but when the second author compared his judgment to the first author's, it was discovered that both authors had reached the same conclusions about which papers should be included and which should be excluded.

## 3. Origin of COVID-19 and its transmission

People with unidentified pneumonia were admitted to hospitals in Wuhan, Hubei Province, China, in December 2019. Experts at the Center for Disease Control discovered that some of them had been exposed to the Huanan Sea food market, where bats, snakes, and other animals were sold (Shereen et al., 2020). Experts discovered on January 7th that it has ~70% similarity to the SARS coronavirus and more than 90% homology with the bat coronavirus. Samples taken from the Huanan fish market were also positive, indicating that the virus originated there (Singhal, 2020). Following the initial few cases in China, several new cases emerged from other regions with no exposure to the sea food and fish market, leading to the conclusion that the virus was most likely spread through human-to-human transmission. More infections were spread because of migration and the gathering of many people around the Chinese New Year. Cases in various Chinese provinces and neighboring countries have been reported in people who arrived after attending a festival in Wuhan. Workers in the health sector who had contact with those patients and their families were later tested positive (Shereen et al., 2020; Singhal, 2020).

Because of the high rate of disease transmission, Wuhan was placed under lockdown on February 23rd, 2020. Patients reported in other countries who had not traveled in a while suggested that local transmission of the virus had occurred in these countries. WHO declared it a pandemic on March 11, 2020, and named the current human coronavirus disease COVID-19. Passengers experiencing symptoms were screened at airports and isolated for testing. Researchers discovered that the virus could spread from patients who have no symptoms, or who are asymptomatic. As a result, the countries that evacuated their citizens from China placed everyone, regardless of symptoms, in a 2-week quarantine and tested them for the virus (Rothe et al., 2020).

**Figure 5:**
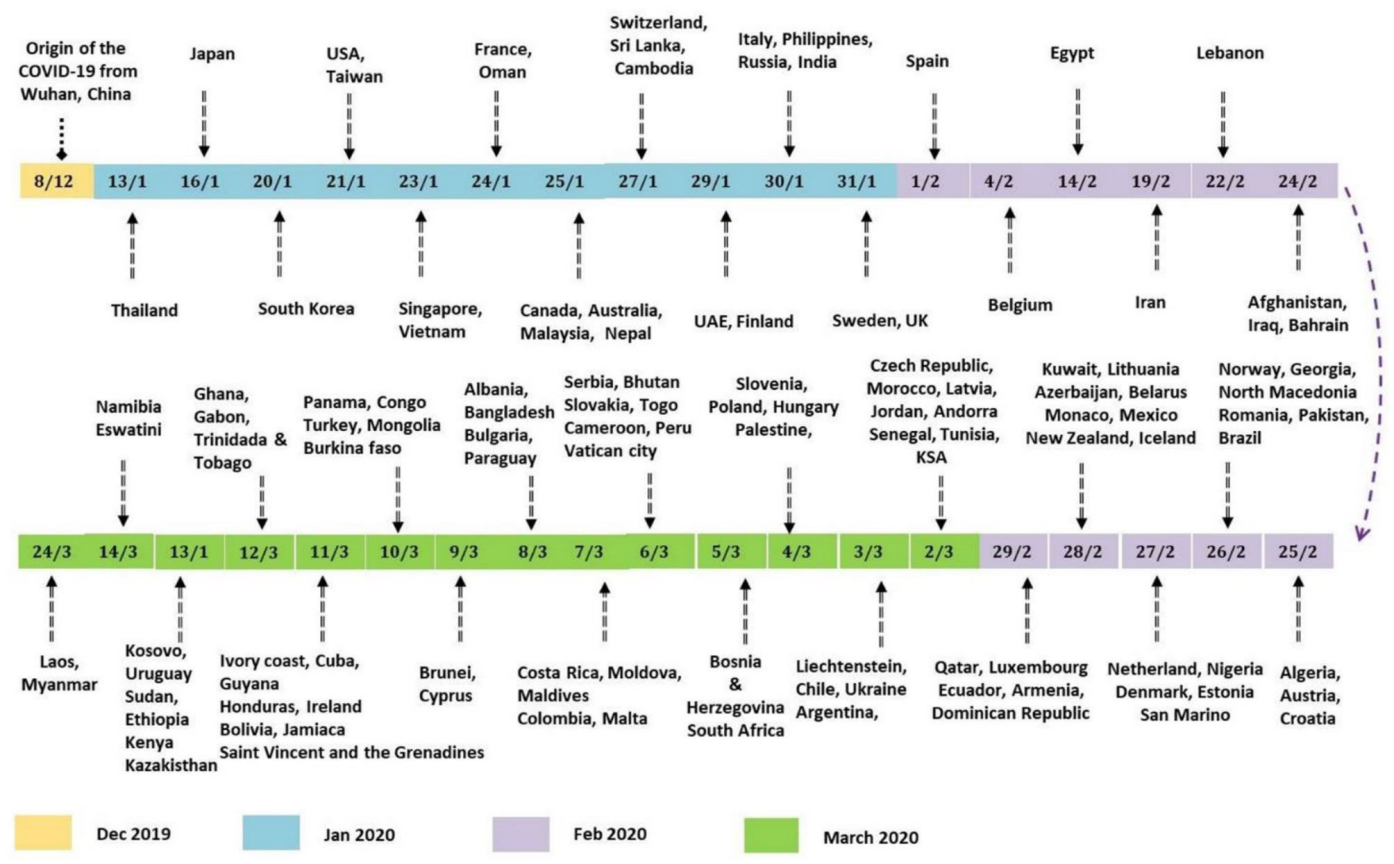
Chronological order of COVID-19 transmission.

A group of Italian tourists was reported to have been infected in India, and later, a patient who flew from Italy contracted the disease along with his six family members. These were the first cases in India in February 2020, which quickly increased in March. To prevent further disease transmission, the entire country was placed under lockdown beginning in March. Those who displayed symptoms were tested and quarantined for 14 days (Pulla, 2020). By the end of March 2020, new cases had decreased in China but had begun to increase rapidly in Iran, Italy, and South Korea, which were later controlled, and the rate of disease spread was eventually reduced in these countries. Following that, in April 2020, a sudden increase in cases was reported from the United States, Britain, India and many other countries. Figure 5 depicts the global distribution of COVID-19 transmission in chronological order (Pulla, 2020).

## 4. Role of artificial intelligence in combating COVID-19

Industry 4.0 witnessed numerous beneficial technologies which may aid in better control and management of the pandemic. These technologies include AI, IoT, Big Data, Virtual Reality, Cloud Computing, Autonomous Robot, 3D Scanning, etc. Amongst them, artificial intelligence facilitates significantly in detecting and diagnosing COVID-19, its symptoms, and other related problems efficiently.

**Figure 6:**
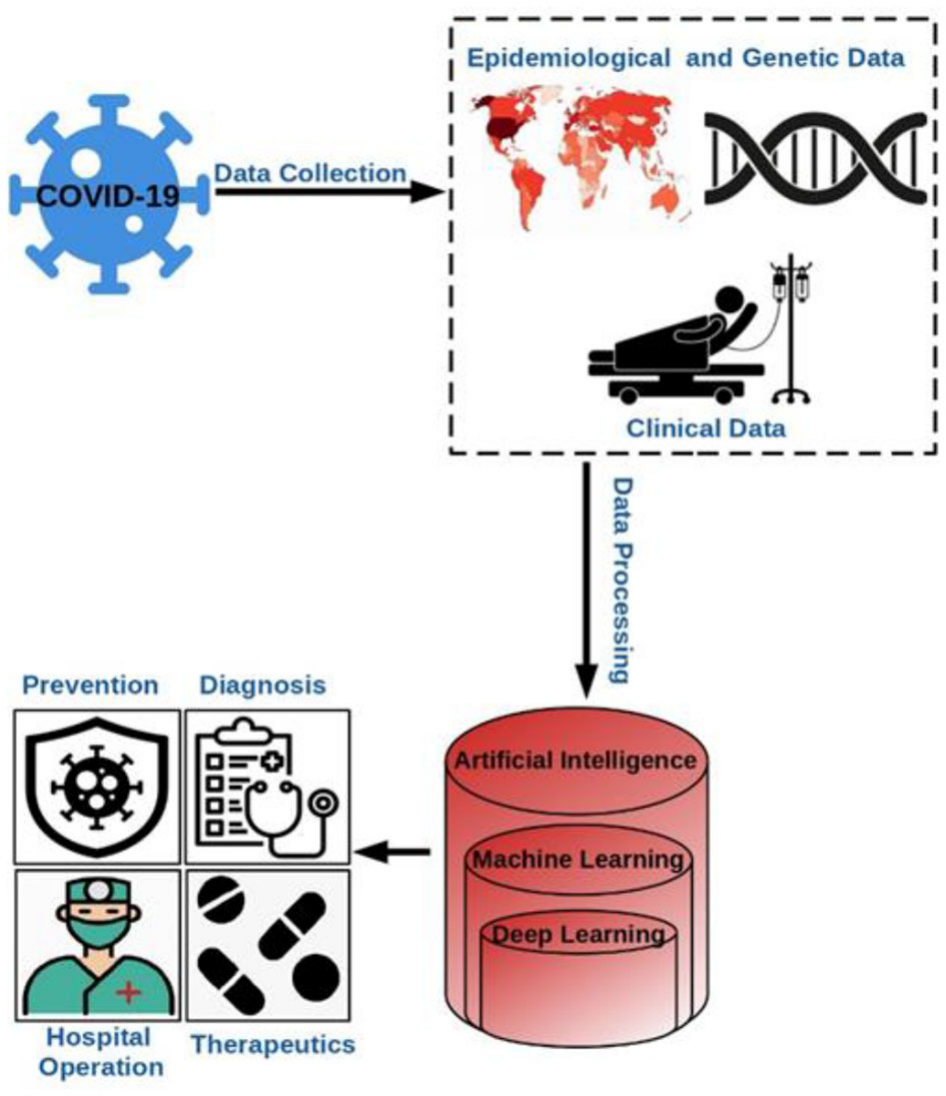
Application of AI to tackle COVID-19.

Considering the historical experiences in the health sector, it has been observed that artificial intelligence plays a crucial role in fighting against the viruses prevalent in those times, which motivated the developers to create applications of AI which reduces human intervention in healthcare amid the pandemic. Artificial intelligence, as of now has been found to be very significant in monitoring, tracing, projection, and development of vaccines. Figure 6 depicts the overall process involving AI that aided in the prevention, diagnosis, hospital management, therapeutics, etc. during the pandemic (Abd-Alrazaq et al., 2020; Alimadadi et al., 2020).

### 4.1. AI in data gathering system for COVID-19

COVID-19 is a transferable disease, as we all know. Minimum human interaction is required to stop this viral disease. COVID-19 data is collected, a monitoring system is developed, and COVID-19 data is visualized using AI-based technologies.

BlueDot is a powerful analytics tool developed in Canada wherein ML and Natural Language Processing (NLP) techniques are employed (Panda, 2020). It was able to detect the onset of COVID-19 and send out watchful warnings. It was also used to send out preventive notices to those cities wherein individuals arrived after January 2020 from Wuhan. The confluence of the user's traces with the traces of infected people was checked using the Global Positioning System (GPS) on smartphones. To keep the data safe, they utilized cryptographic procedures (Arif et al., 2021). After encountering sick people, the app creates early warning indications to assess the danger of infection spreading. Instagram has created “RT. live.”, a COVID-19 tracker which displays state-wise data on virus transmission in the United States. The virus infection's multiplication rate was calculated using data analysis methods (Comba, 2020).

**Figure 7:**
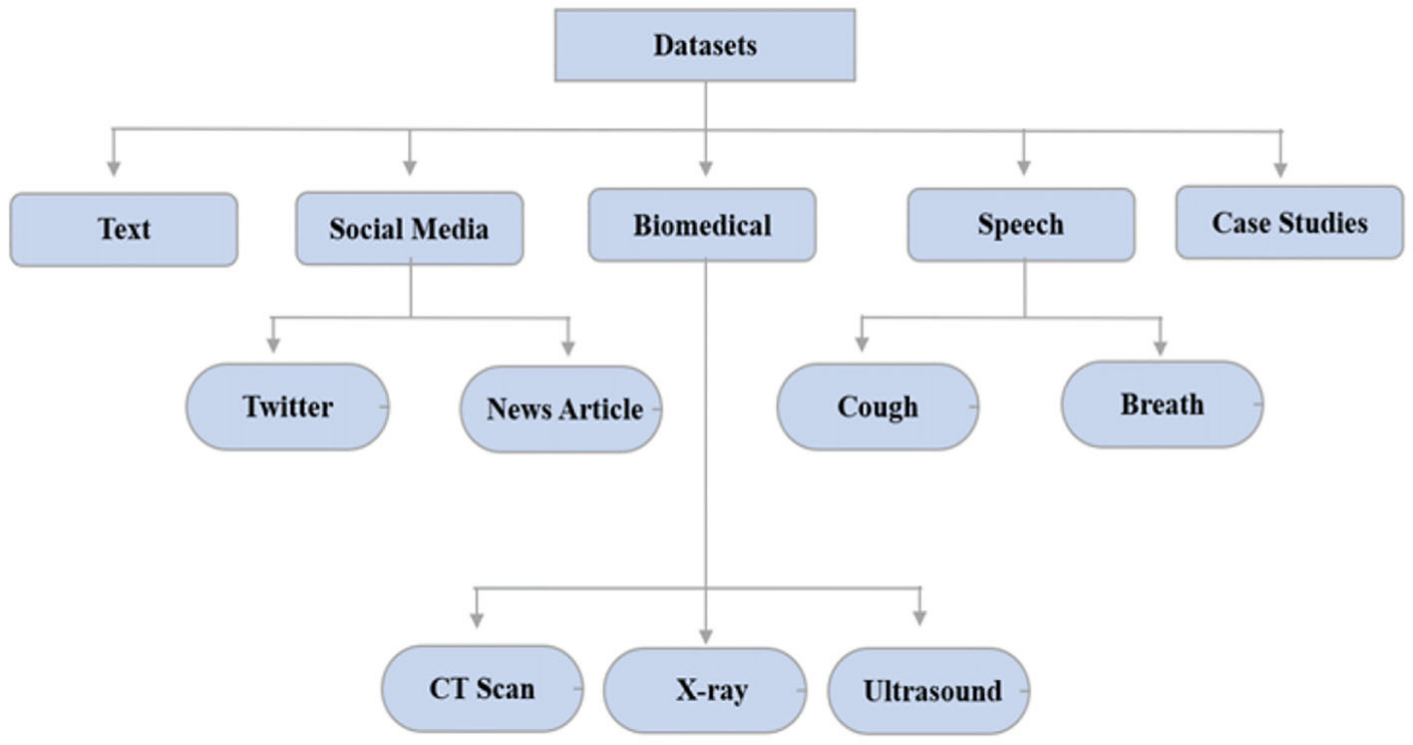
COVID-19 dataset classification.

AI was used to track and forecast COVID-19 infection using data visualization techniques/dashboards. They gave a complete picture of vulnerable patients from every part of the world. Local and global dashboards are the two primary categories of data visualization systems. For a wider population of COVID-19, Johns Hopkins University's Center for System Science and Engineering (JHU-CSSE) created the COVID-19 dashboard. The New York Times provided a virus infection dashboard for several parts of the world as well (Anastassopoulou et al., 2020). The AI tracker of Bing was trained to display diagnosed, cured, and deceased cases from across the world. This filter was used on a specific country for displaying infected people from a certain province. Big data was required for further analyzing medication discovery, risk assessment, infection spread, therapy, and cure of COVID-19 infected patients using Artificial Intelligence approaches. Text, social media, biomedical, speech, and case studies are some of the broad categories for the datasets. The COVID-19 datasets are broadly classified as shown in Figure 7 (Anastassopoulou et al., 2020).

Text datasets included risk variables, non-pharmaceutical interventions, virus infection propagation, incubation period, and environmental stability. Most of the tweets about COVID-19 contained myths and incorrectly classified information about coronaviruses (Abd-Alrazaq et al., 2020; Minaee et al., 2022). To address this issue, various fake news and rumor detection techniques have been proposed. Due to the open-source nature of the COVID-19 chest X-ray dataset, medical images were used to train deep learning or machine learning models (Hughes et al., 2020). According to another study, COVID-19 can also be distinguished by its vocal sounds. The University of Cambridge scholars requested that participants' voice samples be collected to create an open-source cough sample repository. The COVID-19 databases were issued by the World Health Organization and the National Centers for Disease Control and Prevention to track the progression of infection and analyze the influence of preventive measures. COVID-19 contaminated locations can be seen on Johns Hopkins and Git-hub (Andrejeva et al., 2020).

### 4.2. AI in screening patients and diagnosing COVID-19

**Figure 8:**
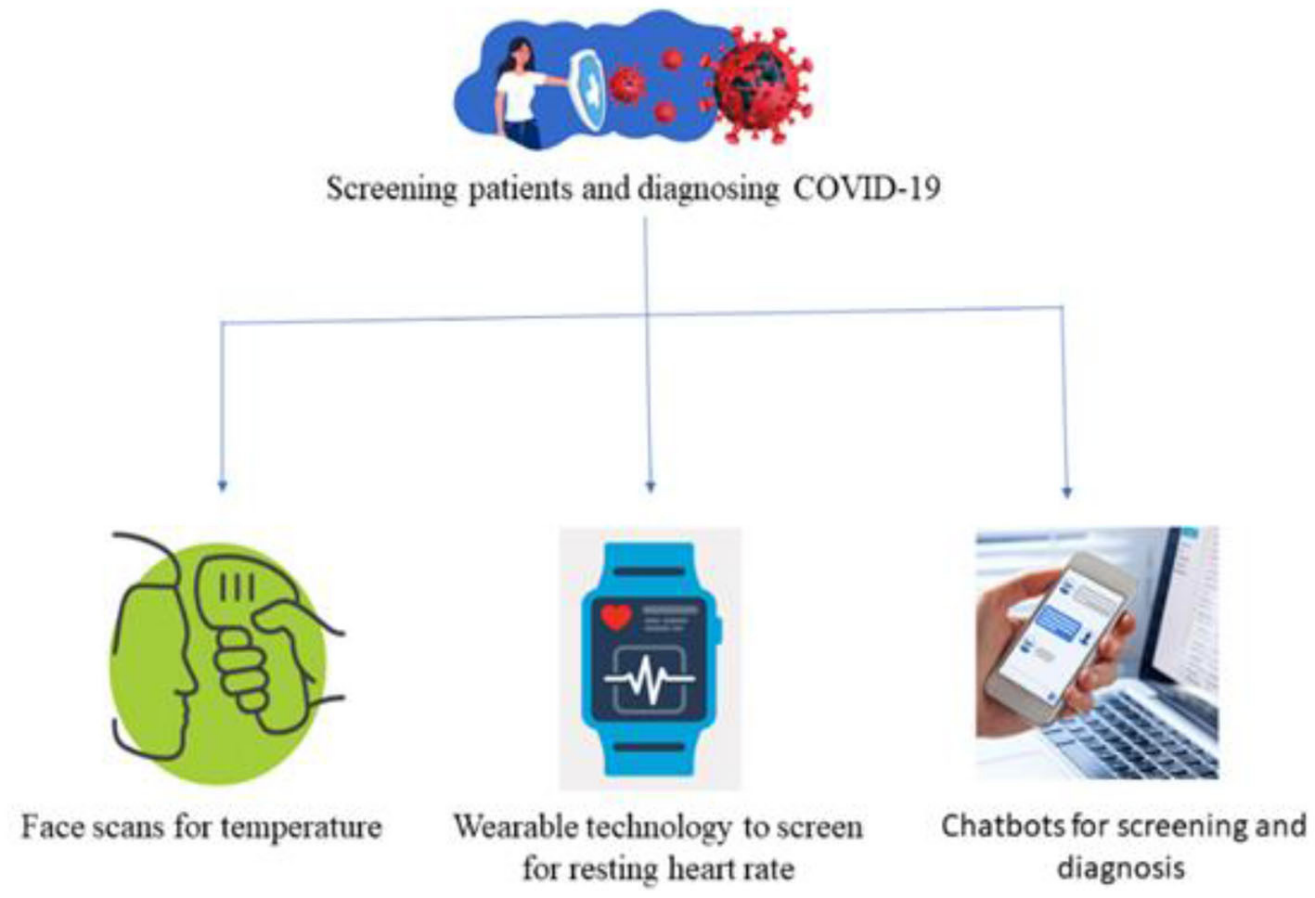
Different ways to screen patients using artificial intelligence.

The diagnosis of a COVID-19 patient is challenging. Each patient is tested individually, which takes a substantial amount of time. For that reason, the medical report must be generated quickly and at a reduced cost. Therefore, COVID-19 patients were also screened using AI-based approaches. There were three main AI-based strategies for screening patients namely face recognition, wearable devices, and virtual healthcare assistant as shown in Figure 8. Applying face scans to determine symptoms, was a potential research field in machine learning to aid in diagnosing COVID-19. Wearable technology, like smart watches, could be used for checking patterns in a patient's resting heart rate. Using chatbots driven by ML to assess patients depending on self-reported complaints (Robitzski, 2020).

Fever in patients was detected using drones/robots equipped with thermal scanners from a safe distance. Baidu, a Chinese company, created infrared cameras to scan crowds for temperature readings (Robitzski, 2020). In a single minute, they can scan hundreds of people. Apple created an AI-powered watch that can detect a patient's temperature along with the heart rate to diagnose their symptoms (Wadhera et al., 2020). Oura Ring, which is designed by OURA enterprise solutions, helps in tracking activity that analyze the patient's onset patterns, progress, and recovery (Nepogodiev et al., 2020).

Similarly, AI-powered Stallion developed a chatbot using natural language processing (NLP) skills as a virtual healthcare agent (Agrebi, 2020). It encourages people to take precautions, monitors their symptoms, and makes recommendations for home quarantine or hospitalization. COVID-19 information was also provided via a virtual assistant developed by Google Cloud. This virtual assistant reacts swiftly to customer questions and provides the best information possible (Sheerman et al., 2020). AskDoc, an AI-enabled doctor video bot, was also developed to offer voice and text replies to COVID-19 questions. Likewise, Facebook created a WhatsApp bot to help people stay up to date on the COVID-19 pandemic (Ardakani et al., 2020).

### 4.3. AI in the early detection of COVID-19

To detect COVID-19 from X-ray and CT images many AI models like, using imaging algorithms have been proposed. X-ray machineries are used to scan the infected body nearly identical to how pneumonia and malignancies are treated. Researchers have put forward a deep convolutional neural network design which was termed as COVID-net where chest X-ray (CXR) images have helped in diagnosing COVID-19. It was developed earlier, as studies which observed those cases, showed irregularities in chest radiography images that were symbolic to those individuals who got infected with COVID-19 (Mahase, 2020). Emara et al. (2021) provided two solutions for an automated COVID-19 diagnostic system. The first was based on transfer learning models that have been pre-trained with chest X-ray scans as input. To achieve this, they employed pre-trained models such as AlexNet, GoogleNet, Inceptionv3, InceptionresNetv2, SqueezeNet, DenseNet201, ResNet18, ResNet50, ResNet101, VGG16, and VGG19. The second method focuses on creating a CNN termed CONV-COVID-net from scratch to identify COVID-19. ResNet models namely ResNet18, ResNet50, and ResNet101 achieved the maximum classification accuracy on the two datasets. According to the results, they achieved 97.67, 98.81, and 100% accuracy using the first dataset, respectively. On the other hand, the reported accuracies for the second dataset were 99, 99.12, and 99.29%, respectively (Chen et al., 2020; Kanne, 2020; Emara et al., 2021).

Using a combination of deep convolutional neural network (CNN) and discrete wavelet transform (DWT) attributes, Mostafiz et al. (2020) suggested an innovative way to identify COVID-19 from a chest X-ray picture. The detection process was completed using a random forest-based bagging strategy. A lengthy experiment was conducted, and the findings depicted that their methodology outperformed existing approaches by more than 98.5% (Mostafiz et al., 2020). The Random Forest (RF) classifier was reported by Sunil et al. for COVID-19 detection. They employed resampling mechanisms to correct the data's imbalance problem. The results of the analysis revealed that accuracy, geometric mean, and f-measure are 0.94, 0.93, and 0.92, respectively. The proposed method offered comparable sensitivity and greater accuracy to deep and transfer learning models, which are computationally costly and less accessible (Kumar and Ratnoo, 2022).

#### 4.3.1. Understanding model choices with Grad-CAM

In the realm of COVID-19 detection from chest X-rays, while much research has focused on creating accurate models, there has been relatively little attention given to making these models explainable. We experimented to bridge this gap by not only developing a classifier for COVID-19 detection but also shedding light on how the model arrives at its conclusions. To achieve this, we integrated Grad-CAM (Gradient-weighted Class Activation Mapping) into our research methodology. Grad-CAM is a technique known for its ability to generate visual explanations. These explanations help us understand the specific areas within the X-ray images that influenced the model's decisions. This step is crucial for making AI-driven diagnostic systems transparent and interpretable, which is vital for clinical applications.

The integration of Grad-CAM allowed us to validate our model's predictions post-analysis and provided valuable insights into the inner workings of the model. By examining Grad-CAM visualizations, clinicians and healthcare professionals can gain a deeper understanding of which regions within the chest X-rays played a significant role in shaping the model's diagnosis. This not only enhances transparency but also facilitates clinical decision-making.

**Figure 9:**
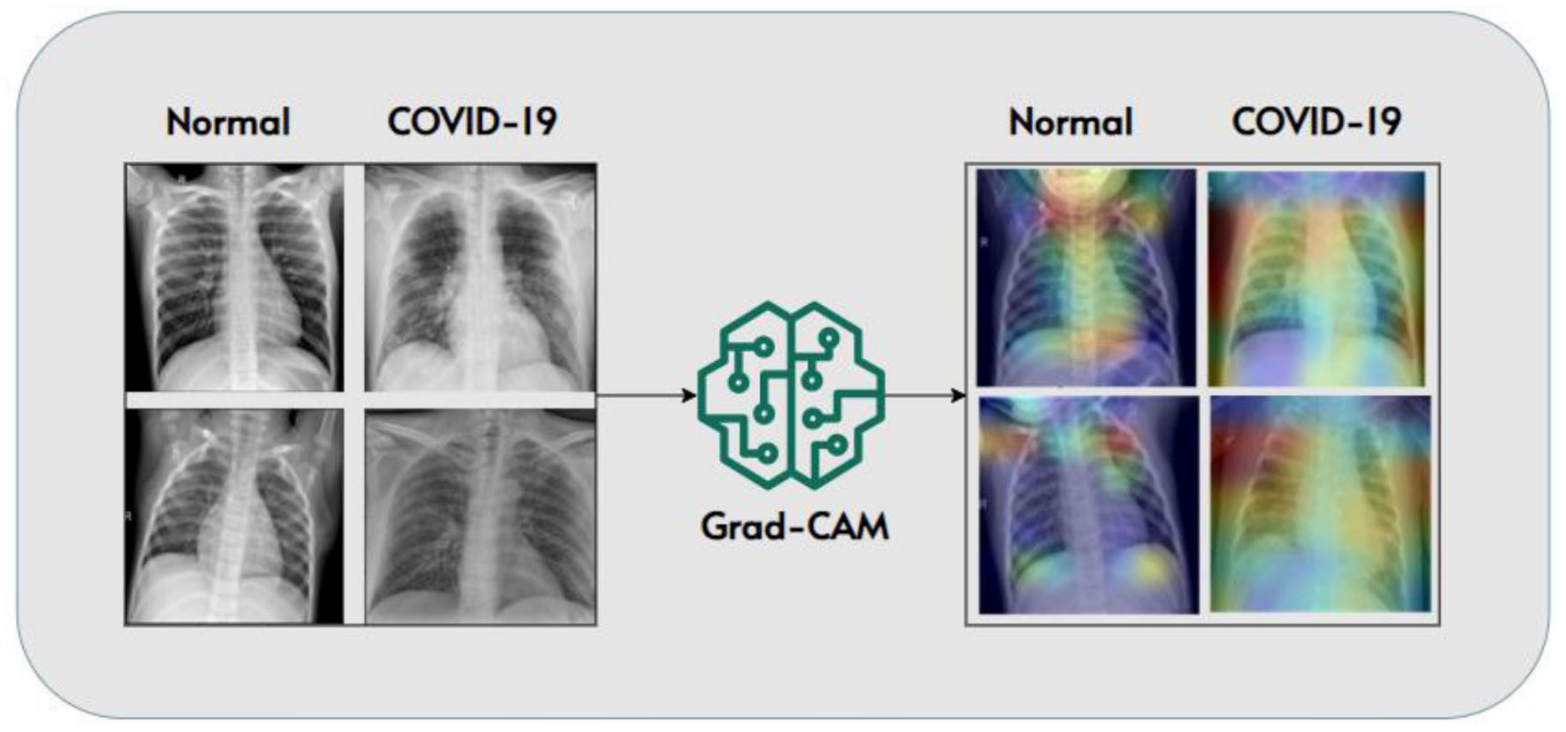
Grad-CAM visualizations highlighting model decision-making (Panwar et al., 2020).

In Figure 9, we showcase a selection of Grad-CAM visualizations. These figures demonstrate how our model identified specific features within the images to distinguish between COVID-19-positive and normal cases. Each visualization highlights the regions of interest identified by the model, helping to demystify the decision-making process. This approach underscores the importance of model transparency and the potential of explain ability techniques like Grad-CAM to make AI-based diagnostics more accessible and trustworthy in the context of COVID-19 and beyond.

**Figure 10:**
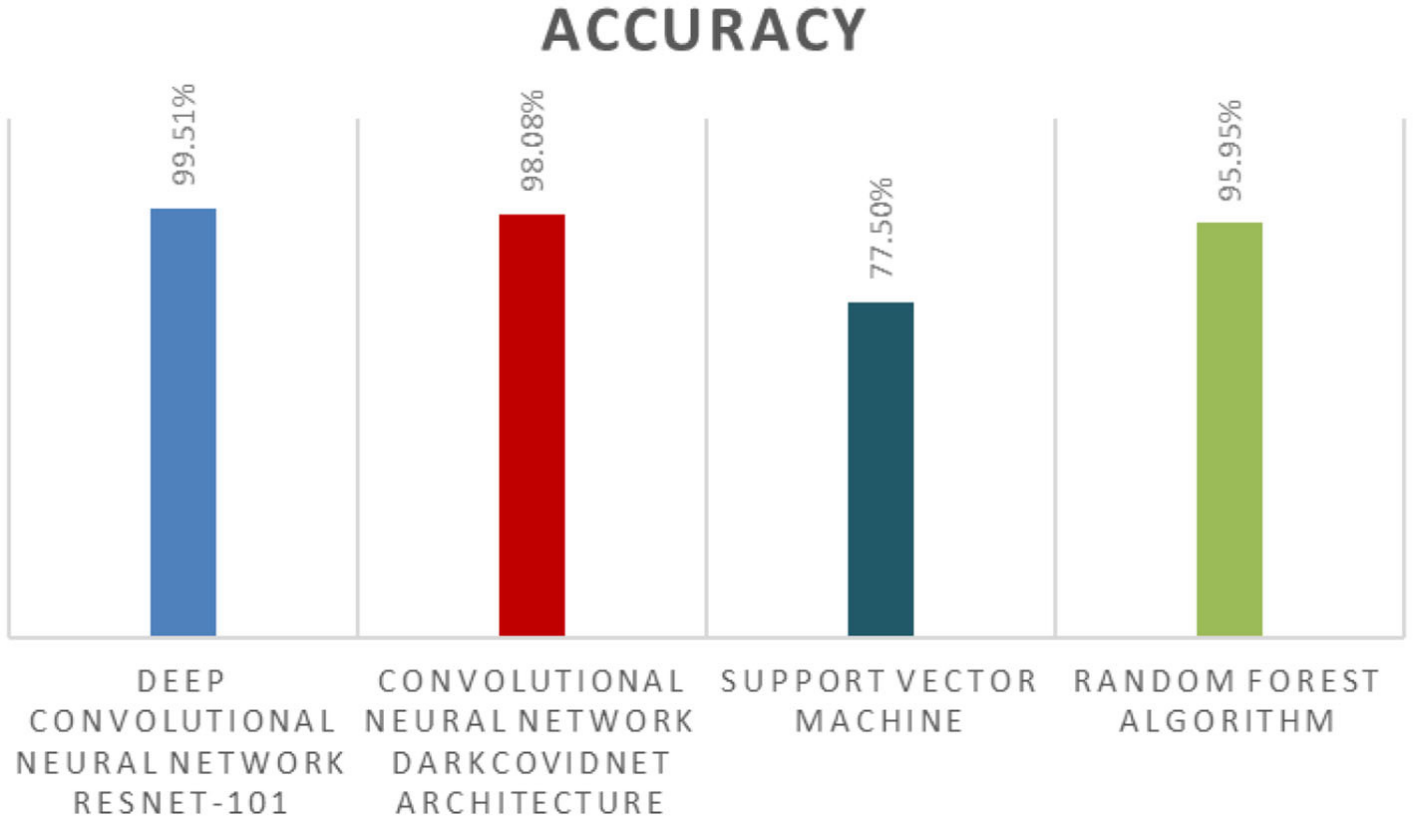
Techniques used in COVID-19 detection with their respective accuracies achieved as given in literature.

Figure 10 compares the average accuracies of different AI algorithms used in diagnosing COVID-19 as mentioned in literature. Deep Convolutional Neural Network ResNet-101 gave the best results achieving an accuracy of 99.51% while Support Vector Machine (SVM) was the least effective with an overall accuracy of 77.50% (Young et al., 2021). A real-time reverse transcription-polymerase chain reaction (RTPCR) was the most extensively utilized technology for diagnosis (Zhu et al., 2020). X-ray and CT scans are two widely recognized radiological imaging methods (Irshad et al., 2023) because RT-PCR has a low sensitivity (60–70%), symptoms could be recognized using radiological imaging (Alam et al., 2021b). For instant results, automated evaluation of CT scans and chest X-rays through ML or deep learning approaches were adopted (Çalli et al., 2021), these methods assisted in speeding up the analysis process considerably (Alam et al., 2021a). Chinese scientists created the InferVISION healthcare application to analyze COVID-19 patients using VIDIA's Clara SDK, it could detect positive instances in a very short span of time. There was a deficiency of clinical expertise in contrast to the number of COVID-19 patients during the pandemic, as a result AI-based tools and methods were used to swiftly diagnose patients (Hasan et al., 2023).

Similarly, Hemdan et al. (2020) built a framework named COVIDX-Net for analyzing X-ray images and tested over 50 X-ray images using seven distinct deep learning models with a 90% accuracy rate. In a similar way, Wang L. et al. (2020) created the COVID-Net model, a deep convolutional neural network (CNN) model for detecting infections in chest X-ray images. The COVID-Net model correctly identified normal, typical pneumonia, and COVID-19 patient 93.3% of the time (Juneau et al., 2023). Ko et al. (2020) used chest CT to build an FCONet model for the classification of COVID-19. VGG16, ResNet50, InceptionV3, and Xception were used by FCONet. FCONet's performance was tested on 3,993 chest CT images. ResNet50 provided a precision of 96.97%. Tan et al. (2021) used a combination of the SRGAN model and VGG16 to diagnose infected individuals using chest CT. The resolution of CT scans was improved using SRGAN. VGG16 was employed to distinguish between the diseased and healthy CT regions, over 275 COVID-19 and 195 normal CT scans. The generated model was validated and produced classification accuracy of 97.87% (Beck et al., 2020).

Likewise, a deep learning model for diagnosing chest X-ray images was created by Jain et al. (2021). The suggested model was built using IncpetionV3, Xception, and ResNeXt. The training and testing accuracies of classification derived from this model were 99 and 96%, respectively. For diagnosing the COVID-19 infection in patients, Islam et al. (2020) used an amalgamation of convolutional neural networks (CNN) and long short-term memory (LSTM). The model's performance was verified on 4,575 X-ray pictures. This model had a sensitivity of 99.3% and a specificity of 99.2%, respectively (Islam et al., 2020).

To evaluate the above results, there are four possible possibilities in the confusion matrix. The number of accurately diagnosed anomalous instances is known as true positive (*Tp*). The number of accurately diagnosed normal instances is known as true negative (*Tn*). The collection of normal instances identified as anomalous cases is known as false positive (*Fp*). The collection of abnormal cases observed as normal cases is known as false negative (*Fn*). Sensitivity (*SN*), specificity (*SP*), accuracy (*AC*), precision (*PR*), mis-classification rate (*MR*), and false positive rate (*FPR*) are all used to evaluate the overall performance of each deep learning classifier (Jensen, 1986) (Table 1).

**Table 1:** Evaluation metrics along with their formulas for calculating the performance of an algorithm.

**S. No**.	**Evaluation metrices**	**Formula**
1	Sensitivity	$SN=\frac{Tp}{Tp+Fn}\times 100$
2	Specificity	$SP=\frac{Tn}{Tn+Fp}\times 100$
3	Accuracy	$AC=\frac{Tp+Tn}{Tp+Tn+Fp+Fn}\times 100$
4	Precision	$PR=\frac{Tp}{Tp+Fp}$
5	Mis-classification rate	$MR=\frac{Fp+Fn}{Tp+Tn+Fp+Fn}$
6	False positive rate	$FPR=\frac{Fp}{Tn+Fp}$

### 4.4. AI in contact tracing of COVID-19 patients

Algorithms applied to the demographic data of multiple cases helped in identifying and tracing COVID-19 patients. Contact tracing ensured identifying individuals with symptoms of the disease and monitored the cluster of cases from spreading the virus further. For this, various mobile applications based on artificial intelligence were developed for tracing the patients as virtual contact tracing is quicker than non-digital methods of tracing. These applications collect the personal data of user and analyze that through AI algorithms to trace if the user is in contact with any COVID-19 patient. Hence, the real-time forecasting model was introduced. It helped in generating predictions of positive cases and deaths in the future by analyzing personal data of the users. The model also checked whether the user was likely to spread the disease in particular regions which in turn helped in taking safety measures in those areas (Beck et al., 2020).

For example, the British government tested an app that used Bluetooth protocol to locate other smartphone owners that are in close vicinity to one another. As a result, a person who was not affected but was in close proximity to someone who had COVID-19 symptoms could be notified. In addition to contact tracing, Nieva, Melek, and a group of Waterloo Engineering students are developing software algorithms based on smartphone's Bluetooth data for grouping persons within a community. They're also working on wearable Bluetooth devices to increase the app's accuracy even more. Through the assessment of specific vital signs, the AI algorithms will increase contact-tracing accuracy and real-time observation of the recovery progress (Richardson et al., 2020).

Table 2 shows several infected countries that were efficient in developing contact tracing applications based on machine learning and artificial intelligence. They created a digital contact tracing method with a mobile application, integrating various technologies like Bluetooth, GPS, contact information, network-based API, system physical address and mobile tracking data. According to studies, over 36 countries have effectively implemented digital contact tracing using centralized, decentralized, or a mixture of both systems to reduce work and enhance the efficacy of traditional healthcare diagnostics procedures (Tang et al., 2021). All these apps are intended to capture personal data, which will be analyzed by machine learning and artificial intelligence (AI) algorithms to track down an individual person who is susceptible to the virus because their latest contact sequence (Chakraborty and Ghosh, 2020; Ubaid et al., 2021).

**Table 2:** Different contact tracing applications used by infected countries.

**Location tracking technology**	**Countries and contact tracing apps**
Bluetooth	Australia (*COVIDSafe*), Austria (*Stopp Corona*), Bahrain (BeAware Bahrain), Czech Republic (*eRouska*), Estonia (*Estonia's App*), Finland (*Ketju*), France (*StopCOVID*), Germany (*CoronaApp*), Hungary (*VirusRadar*), India (*Aarogya Setu*), Ireland (*HSE COVID-19 App*), Italy (*Immuni*), Latvia (*Apturi COVID*), Malaysia (*MyTrace*), Mexico (*COVIDRadar*), North Macedonia (*StopKorona*), Norway (*smittestopp*), Poland (*ProteGO*), Qatar (*Ehteraz*), Saudi Arabia (*Corona Map*), Singapore (*TraceTogether*), Switzerland (*SwissCOVID*), Turkey (*Hayat Eve Sigar*), UAE (*TraceCOVID*), UK (*NHS COVID-19 App*)
GSM	Bulgaria (*ViruSafe*), Bahrain (*BeAware Bahrain*), China (*Health code*), Cyprus (*CovTracer*), Iran (*Mask.ir*), Norway (*smittestopp*), Qatar (*Ehteraz*), Turkey (*Hayat Eve Sigar*)
GPS	Cyprus (*CovTracer*), Columbia (*CoronApp*), Ghana (*GH COVID-19*), Iceland (*Ranking C-19*), Jordan (*AMAN App*)

### 4.5. AI in speeding up drug development

It is evident that AI aids in increasing the speed of the drug testing process which involved a great deal of time initially. Since it boosts diagnosis prediction and drug development, as a result, it helped in producing the vaccine much faster. A molecule transformer-drug target communication design was introduced by scientists that can help in finding an anti-viral drug to treat coronavirus (Laudanski et al., 2020). The model compared with an application named Autodok Vina and applied algorithms on some existing drugs approved by the FDA. It was found that antiretroviral drug Antazanavir and Remdesivir were beneficial for COVID-19 patients. There is a low-risk factor to use existing drugs and moreover it is a less expensive process comparatively. Consequently, AI was noted as one of the most significant technologies for enhancing the drug development process and finding accurate results (Yigitcanlar et al., 2020).

Several research laboratories worked continuously to create COVID-19 vaccines and medicines during this epidemic. They utilized artificial intelligence to find new vaccinations or repurpose old ones (Kühl et al., 2020). The most potential virus-host protein-protein interactions for HIV as well as for H1N1 have been effectively predicted using ML models trained on datasets of protein, considerably decreasing the work necessary to create a comprehensive map of the virus-host interactome. AI may be used to screen existing medication compounds and determine whether or not they are effective in combatting coronavirus. South Korea and USA employed a system based on AI for discovering the “Atazanavir” medicine that was repurposed to treat COVID-19 (Liu et al., 2020). Benevolent AI researchers discovered the medications “Baricitinib” and “Myelofibrosis” for curing COVID-19 (Hossain et al., 2020).

### 4.6. AI in the prediction of future pandemics

As we cast our gaze beyond the ongoing COVID-19 pandemic, it becomes increasingly critical to explore how Artificial Intelligence (AI) can play a proactive role in preventing and mitigating future pandemics, including emerging threats like Dengue, Salmonella, and Monkeypox. AI offers significant potential to strengthen our preparedness and response strategies. Trying to predict if a variant of influenza will spread from person to person via zoonotic transmission can assist doctors and other experts to plan of time and prepare for pandemics that could occur. Influenza A, for instance, is predominantly found in birds, but it has the potential to spread to humans (Louten, 2016).

The researchers detected possible zoonotic influenza strain with great precision using machine learning. Many AI/ML-based models have been successful in determining the future of pandemics, such as infection or transmission rate, according to the literature. As a global ML-based prediction method, Ramesh et al. proposed the probability of a COVID-19 recurrence. Exponential Smoothing (ES), Least Absolute Shrinkage and Selection Operator (LASSO), Support Vector Machine (SVM), and four typical prospective predictions, such as Linear Regression, were all identified as risk factors for COVID-19 in this study. The study estimated the number of newly infected COVID-19 patients, death rates, and COVID-19 recovered cases in the next 10 days (Mojjada et al., 2020). Similarly, other researchers have also proposed different AI/ML based modeling techniques to forecast a pandemic such as COVID-19. A comparison of existing works on COVID-19 prediction methodologies is depicted in Table 3.

**Table 3:** Comparative study of existing works on COVID-19 prediction methodologies.

**References**	**Predictive methods (algorithms)**	**Prediction span**	**Data source**	**Remarks**
Devaraj et al. (2021)	ARIMA, LSTM, Stacked LSTM, Prophet for time series forecasting	30, 60, and 90 days ahead	Center for systems science and engineering (CSSE) at Johns Hopkins University (JHU), world weather, Wikipedia pages	SLSTM performed better than other models utilized in the study
Kirbaş et al. (2020)	ARIMA, non-linear auto-regression neural network (NARNN), (LSTM)	14 days ahead	European Center for Disease Prevention and Control	The LSTM model has higher mean absolute percentage error (MAPE) values than the other models
Arora et al. (2020)	Deep LSTM/stacked LSTM, convolutional LSTM, bi-directional LSTM	Daily and weekly	Ministry of Health and Family Welfare (India)	Bi-directional LSTM produces better outcomes with less inaccuracy than other models
Zeroual et al. (2020)	Recurrent neural network (RNN), LSTM, Bi-directional LSTM, variational auto-encoder (VAE)	17 days ahead	Center for Systems Science and Engineering (CSSE) at Johns Hopkins University (JHU)	VAE surpassed other models in predicting the pandemic
Shahid et al. (2020)	ARIMA, support vector regression (SVR), LSTM, Bi-LSTM	48 days ahead	Harvard University	Bi-directional LSTM dominated other models with better *R*^2^ scores
Alzahrani et al. (2020)	ARIMA, auto-regressive moving average (ARMA)	1 month ahead	Saudi Arabia government website	ARIMA outperforms ARMA, moving average (MA), and auto-regressive (AR) models
Ogundokun et al. (2020)	Linear regression model	8 days ahead	National Cooperative Development Corporation (NCDC) website	The model achieved 95% accuracy on the dataset
Tomar and Gupta (2020)	LSTM	30 days ahead	JHU CSSE	LSTM achieved a 90% accuracy rate in predicting COVID-19 confirmed patients
Car et al. (2020)	Multilayer perceptron (MLP) artificial neural network (ANN)	30 days ahead	JHU CSSE, Environmental Systems Research Institute (ESRI) Living Atlas Team, JHU Applied Physics Laboratory (APL)	Higher level of accuracy for reported cases
Hawas (2020)	Recurrent neural network (RNN)	30 and 40 days ahead	JHU CSSE	60.17% accuracy achieved on the dataset utilized for the study
Alassafi et al. (2022)	RNN, LSTM	7 days ahead	European Center for Disease Prevention and Control	LSTM performed better than RNN with an accuracy of 98.53%
Petropoulos et al. (2022)	Simple time series model with multiplicative trend	10 days ahead	JHU CSSE	The model performed just as well as other popular models but might be more suitable when less data is available
Ketu and Mishra (2022)	CNN-LSTM, LSTM, and ARIMA	40–50 days ahead	Ministry of Health and Family Welfare (India)	The CNN-LSTM hybrid model performs better than other models utilized in the study
ArunKumar et al. (2022)	SARIMA, ARIMA, GRU, LSTM and various hybrid combinations of the same	60 days ahead	JHU CSSE	SARIMA based models fared better for India, Russia, Peru, Chile, and the UK for confirmed cases. For Brazil, the ARIMA model performed better. The LSTM model fared better for Mexico and Iran, whereas the GRU model did better for the USA and South Africa
Naeem et al. (2023)	ANN, KF, LSTM, and SVM	21 days ahead	JHU CSSE	LSTM performs better than other models

Although more research is needed to develop models for predicting direct transmission, the knowledge of which strains of influenza are most probable to leap is a vital first step in planning for the next pandemic. In the future, AI systems could be developed for pandemic prevention and response, with its capabilities spanning early disease detection, predictive modeling, rapid vaccine development, efficient resource allocation, contact tracing, targeted public health communication, and genomic analysis. More patient data exchange, as well as ML approaches that would enable models to be built whilst limited data is accessible, would help. Few-shot learning, in which AI can understand patterns from a limited number of findings, along with transfer learning, in which an AI that has successfully received training to achieve one task could be swiftly changed to perform some another similar task, are examples of potential gains. Such proactive approaches align well with the evolving landscape of infectious disease management and underscores AI's pivotal role in safeguarding global public health in the future. However, there is still more work to be accomplished. In addition, we can also explore the use of large language models, namely ChatGPT or Google Bard in case of any future pandemic outburst (Sohail et al., 2023a).

AI language models like ChatGPT hold significant potential for future pandemics. They can play a vital role in disseminating accurate information to the public, answering queries about prevention, symptoms, testing, and vaccination, thus aiding in education. Additionally, AI can be leveraged to power crisis helplines and mental health support, offering much-needed emotional assistance and guidance (Sohail et al., 2023a,b). ChatGPT's language translation capabilities can ensure that critical information reaches non-English speakers and diverse populations. Monitoring public sentiment through AI can guide authorities in tailoring responses effectively. Furthermore, AI can assist in detecting and correcting misinformation, aiding in research and drug discovery by sifting through vast amounts of scientific literature, supporting remote patient monitoring, engaging citizens in policy discussions, designing awareness campaigns, and enhancing healthcare worker training.

## 5. Discussion and open issues

In the previous section a detailed AI driven algorithms and approaches have been discussed. In addition to this, the datasets that have been exploited to further the research and experiments for establishing an AI based solution to the pandemic has also been studied. However, the studied approaches have some limitations as well. For example, these algorithms are not a sure diagnostic tool to identify the patients infected with SARS-CoV-2 virus nor a complete treatment for the patients. To design an integrated tool that can help healthcare officials and patients, a robust mechanism is needed. Moreover, identifying the algorithm which could perform better in the established approaches based on various deep learning and related mechanism would be pivotal for creating options for such pandemic. For example, according to Devaraj et al. (2021), in Auto-Regressive Integrated Moving Average (ARIMA), Long Short-Term Memory (LSTM), Stacked LSTM (SLSTM), and Prophet for time series forecasting, SLSTM is superior in accuracies than other models.

Furthermore, not only identifying patients with similar symptoms or trying to provide an AI based early treatment would be sufficient in near future, rather an approach needs to be devised that can also resist the spread and outbreak of such a pandemic in near future. This we can see from the recent another global emergency “Monkeypox”. In this context, social media can serve as a game changer (Jena, 2021). If of the development various AI algorithms like NLP and sentiment analysis techniques, and recent data mining development can be exploited further to achieve this goal. Amir and Aziz (Hussain and Sheikh, 2021) has suggested how deep learning and recent advancement in AI approaches can be adequately utilized. For instance, social media booms can leverage the information propagation regarding pandemic. It can thus be used to inform its users regarding safety parameters and measures to avoid pandemic spread. Moreover, it can also be helpful in capturing real-time public opinion and hence can serve better in generating a consensus on the solutions to such global emergencies like COVID-19 and Monkeypox, etc. Like, with the help of sentiment analysis and state-of-the-art techniques in NLP, the public perception on a certain event can be assessed (Aslani and Jacob, 2023). These events may include finding out the opinion of a common man on vaccine delivery, and exploring their responses to the safety measures and their feedback on how they are been treated by medical practitioner at hospitals, etc.

Similarly, SOS can be alerted and provided across these social platforms. Many of us have witnessed how a quick SOS on Twitter helps in identifying someone in emergency and how the health officials and social service volunteers could save many lives (Catalan-Matamoros et al., 2023; Nayyar et al., 2023; Shakibfar et al., 2023; Yuan et al., 2023). More precisely, AI have been vital during COVID-19 and helped a lot in various dimensions. However, still it faces many challenges and research issues which must be addressed and handled by the research community to prevent any catastrophe by similar pandemic in near future.

## 6. Conclusion

This pandemic has had negative consequences across the world. In this paper, we have reviewed the studies on COVID-19 and the significance of artificial intelligence in combating this pandemic. Artificial intelligence has been observed to have a tremendous impact during this time. Adoption of new technology by professionals, researchers, and healthcare workers has increased rapidly all over the world. AI helps in reducing human intervention in almost every sector and especially in the healthcare sector. It is significant in monitoring, detection, tracing, projection, and development of the vaccine for COVID-19. Telemedicine has been adopted as an essential means to maintain the health system amidst the pandemic. The optimal AI model aids in working order within the medicinal field and helps in attaining more efficiency, more prominent accuracy, enhanced productivity, and productive outreach during this COVID-19 pandemic. It is envisaged that the study shall serve medical professionals and researchers in subjugating the obstacles encountered while examining COVID-19 data.

## Author contributions

FF: Conceptualization, Data curation, Formal analysis, Investigation, Methodology, Validation, Writing—original draft, Writing—review & editing, Visualization. SS: Conceptualization, Formal analysis, Investigation, Methodology, Project administration, Resources, Supervision, Writing—original draft, Writing—review & editing, Visualization, Data curation. MAl: Investigation, Resources, Validation, Visualization, Methodology, Writing—original draft. SU: Investigation, Resources, Validation, Visualization, Writing—original draft. S: Investigation, Resources, Validation, Visualization, Writing—original draft. MAs: Investigation, Resources, Validation, Visualization, Writing—review & editing. DM: Funding acquisition, Project administration, Resources, Writing—review & editing.
